# Tin Foil as a Filling

**Published:** 1884-02

**Authors:** 


ARTICLE V.

### TIN FOIL AS A FILLING.

(Extract from Transactions of Ohio State Dental Society.)

Dr. Berry took the chair.

The President: I have seen operations which were performed twenty or thirty years ago with tin foil, still in good condition. I thought if tin foil was good then it would be much better now. I have used it quite successfully. But I thought it might be improved. I cut a strip of tin foil about one-sixteenth of an inch in width; I then take about one-fourth of a sheet of gold foil, lay it on a napkin, place the strip of foil at the edge of gold foil; then I take the white card that comes in the book of gold, place the edge against the strip of tin and with a thin spatula fold the gold over the tin four or five times, then cut into pellets. I have *annealed those pellets gently* and get gratifying results. I can get both adhesive and cohesive qualities which are deficient in many filling materials ; it also gives more density. I find it invaluable in sensitive teeth, where it is almost impossible to drill retaining pits ; if you have but a slight concave surface, you can place the tin foil in it and it will stay to its place with greater adaptation and less recoiling than any other filling known. I also find that in order to make any of the cement fillings (I mean oxychlorides or oxyphos) at all durable, it is important to fill first with the tin foil down to or a little below the margin of the gum, then add your cement, and your filling is worth ten fold more when made in that way.

Dr. Butler : I would like to show some points that I regard as valuable in using this particular filling, because the manipulation of it requires an instrument of peculiar construction to work FREE. Tin foil is one of the oldest substances used in dentistry. It is unnecessary for me to state its great value, but with all that I venture to say that there are but few men who get as good results from tin as might be done. I made an effort to secure the presence of an old practitioner, the first prominently successful dentist in this State, but did not succeed. From his hands I have seen the best results I know of with tin foil. For years I endeavored to have him show me how he secured such results. He would say, " you can put them in just as good." But having seen him go through the manipulation, I was satisfied where I had made the mistake. He claimed that tin should be produced with cohesive property somewhat like gold, and he labored with different manipulators to secure that object. There is now in the market a filling far superior to what we had some years ago. This fibrous preparation has given us a very admirable quality of cohesiveness ; it is a firm, solid mass when well packed. But it can deceive us. The cavity may be filled but not solid or tight ; it requires great force to make it hard, and the light may not be sufficient for you to see these points. The peculiar softness of it, whether the serration be coarse or fine, renders it liable to become clogged, and then it don't work at all. I will speak of the peculiarity of these serrations. They are on the side as well as on the end; the angle of the serrate is cut, instead of being right against the filling it laps down and makes the serration stand downward. It requires a good deal of force to carry it down and make a solid mass. You can just take and cut it down like a block of tin when thoroughly packed to use it in conjunction with gold. It has been claimed that it adheres to this tin as readily as to itself. Now, if you haven't been deceived in that you will be. I have. That is an over-statement. Nevertheless it is a good thing. You drive it into the cavity, straight down, and commence and build your gold as if you hadn't it there at all. It is a very nice thing for a base to support your gold filling upon. You have the gold sustained by the tin, the filling sustains the tooth, the tooth the filling. I consider it a very valuable thing. I consider this one of the advanced means to the end. Here are a couple of instruments. These are not typical instruments —I would prefer a larger handle. These mallet instruments are smooth in the serrations and work freely, being stoned out smooth.

I have filled a good many front teeth, placing a lining of gold against the labial wall, using that for a base, and they have been saved longer than by a gold filling. And I think there is a quality about this that you can drive it hard, that is, if the instrument be not too much wedge-shaped, and get into close proximity to the cavity without danger of bursting.

I prefer the thickest sheets made. If using for a small cavity, I would cut it in little strips; it has a fibre in it, and if you cut across you will have these little short pieces. Cut it one way and it breaks easily; the other way it is pretty strong. That breaking up can be avoided by the manner in which you cut it.

A Member: It is more reliable in bad shaped cavities than gold?

Dr. Butler: Take a bad shaped cavity and drive a mass in—it has no recoil about it—and there is not as much danger it being thrown out as gold. In a large cavity I sometimes take this and put a gold roof on it. They work just as well. I do not know that I can make it any plainer. I have a preparation here—it is not new, but the formula has been changed—that I call sticking wax. If you have a dam on and nick a hole through, raise it up, stick on a little of this wax hot, and go on and finish your operation without stopping; the wax neither gets soft nor hard. The heat of the mouth makes it very pliable. It will stick tight to any dry surface. The formula of this was given by Dr. C. F. Wheeler. Take seven ounces of gum damar to four ounces of pure white wax. To this add one drachm of Canada balsam; there is no paraffine in it. Melt the gum damar first, then add the wax and balsam; then spread out to cool. It is also a very valuable thing if you want to put together any neat piece of mechanical work while adjusting it.

Dr. Templeton: The first thing I would say is that this fibrous material has almost superseded amalgam in my practice. You know I live in Pittsburgh, and that is saying a good deal, for Pittsburgh has always been a stronghold for amalgam. Some of you remember a certain man who used to live in Ohio wanted, a number of years ago, to sell a patent right for amalgam, but he didn't call it that. He called it the "Royal Mineral Succedaneum." Now, in regard to the use of fibrous material. It is about sixteen months since I commenced using it, and I do like to use that material. We can preserve the teeth better with that filling than we can with amalgam. I have never seen amalgam that wouldn't shrink. If there is anything that a dentist uses that will injure his reputation among people, it will be amalgam. You talk about elevating the profession; if you want to elevate it stop the use of amalgam. About fifteen years ago I was living at New Castle, Pennsylvania. At that time everybody wanted their teeth filled with silver, and in some cases I put in amalgam ; but there were so many failures that I didn't want to use it any more. I then used tin foil, and some of them are standing to-day. There is something about tin foil that is preservative. I find less decay in the neighborhood of tin foil than any other filling.

Dr. E. J. Waye : Thus far we have nothing but praise of the felt foil. On this occasion I feel constrained to take the opposite side. I had heard such unqualified praise of this material from men who are presumed to be judges of its merits that I was prepared to adopt it without my usual amount of experimentation. In one particular place in a certain kind of cavity its use was warmly commended, and that was in deep and inaccessible crown and approximate cavities, where it was claimed it could be perfectly adapted without difficulty to the cervical and side walls, more perfectly than gold and with far less labor and care make a perfect joint. In my hands I found to my disappointment the statement did not hold good, and that, unless more care and time was used in working it, what was supposed to be a perfect joint would be found a failure. In my judgment this new material ranks as a tin filling with crystal gold in gold. With either material excellent fillings may be made, but that felt foil has no greater cohesive quality than tin foil, requires more time and care to adapt it to the walls of cavities than tin foil, is ten times as expensive, and is in no particular superior to it as a material for filling teeth.—*Transactions of the Ohio State Dental Society.*

